# Identification of Renoprotective Phytosterols from Mulberry (*Morus alba*) Fruit against Cisplatin-Induced Cytotoxicity in LLC-PK1 Kidney Cells

**DOI:** 10.3390/plants10112481

**Published:** 2021-11-17

**Authors:** Dahae Lee, Seoung Rak Lee, Bang Ju Park, Ji Hoon Song, Jung Kyu Kim, Yuri Ko, Ki Sung Kang, Ki Hyun Kim

**Affiliations:** 1 College of Korean Medicine, Gachon University, Seongnam 13120, Korea; pjsldh@gachon.ac.kr; 2 School of Pharmacy, Sungkyunkwan University, Suwon 16419, Korea; seungrak@princeton.edu; 3 Department of Electronic Engineering, Gachon University, Seongnam 13120, Korea; sooyong1320@gachon.ac.kr; 4 Jeju Institute of Korean Medicine, Jeju 63309, Korea; jhsong@jikom.or.kr; 5 School of Chemical Engineering, Sungkyunkwan University, Suwon 16419, Korea; legkim@skku.edu; 6 Department of Biological Chemistry and Molecular Pharmacology, Harvard Medical School, Boston, MA 02115, USA; koyr0120@gmail.com

**Keywords:** mulberry, *Morus alba*, phytosterols, LLC-PK1, nephrotoxicity, MAPKs

## Abstract

The aim of this study was to explore the protective effects of bioactive compounds from the fruit of the mulberry tree (*Morus alba* L.) against cisplatin-induced apoptosis in LLC-PK1 pig kidney epithelial cells. *Morus alba* fruit is a well-known edible fruit commonly used in traditional folk medicine. Chemical investigation of *M. alba* fruit resulted in the isolation and identification of six phytosterols (**1**–**6**). Their structures were determined as 7-ketositosterol (**1**), stigmast-4-en-3β-ol-6-one (**2**), (3β,6α)-stigmast-4-ene-3,6-diol (**3**), stigmast-4-ene-3β,6β-diol (**4**), 7β-hydroxysitosterol 3-O-β-d-glucoside (**5**), and 7α-hydroxysitosterol 3-O-β-d-glucoside (**6**) by analyzing their physical and spectroscopic data as well as liquid chromatography/mass spectrometry data. All compounds displayed protective effects against cisplatin-induced LLC-PK1 cell damage, improving cisplatin-induced cytotoxicity to more than 80% of the control value. Compound **1** displayed the best effect at a relatively low concentration by inhibiting the percentage of apoptotic cells following cisplatin treatment. Its molecular mechanisms were identified using Western blot assays. Treatment of LLC-PK1 cells with compound **1** decreased the upregulated phosphorylation of p38 and c-Jun N-terminal kinase (JNK) following cisplatin treatment. In addition, compound **1** significantly suppressed cleaved caspase-3 in cisplatin-induced LLC-PK1 cells. Taken together, these findings indicated that cisplatin-induced apoptosis was significantly inhibited by compound **1** in LLC-PK1 cells, thereby supporting the potential of 7-ketositosterol (**1**) as an adjuvant candidate for treating cisplatin-induced nephrotoxicity.

## 1. Introduction

Cis-diamminedichloroplatinum II (cisplatin) is one of the most common platinum chemotherapeutic agents used for the treatment of many types of solid tumors [1]. In more than 30% of patients taking cisplatin, a variety of side effects, including allergic reactions, ototoxicity, myelotoxicity, nephrotoxicity, and gastrotoxicity, have been reported [2]. Of these side effects, nephrotoxicity is a dose-limiting one that makes patients unable to continue cisplatin treatment [3]. Cisplatin can seriously damage the S3 segment of the proximal tubules, causing kidney dysfunction [4]. Forced diuresis using mannitol, magnesium supplementation, and kidney-protective therapeutic approaches using enzymes and compounds that can help treat or prevent cisplatin-induced nephrotoxicity was reported [5]. In addition, the effects of plant extracts and plant-derived natural products on cisplatin-induced nephrotoxicity were studied [6]. However, the detailed molecular mechanisms underlying their protective effects remain unclear. In previous studies using kidney cells, treatment with cisplatin (16–300 μM) induced cell death and activated cellular signaling pathways, including p53, mitogen-activated protein kinases (MAPKs), and caspases [7,8], which can be molecular targets for the mechanism of nephroprotection.

The mulberry tree (*Morus alba* L.), also known as white mulberry, belongs to the family Moraceae. *Morus alba* fruit is a well-known edible fruit commonly used in traditional folk medicine to improve diabetes and eyesight [9]. Its leaves are also consumed as a fodder for silkworms (*Bombyx mori* L.) and used in health products such as tea and beverages [10]. In previous studies on *M. alba*, extracts from its fruit have exhibited pharmacological activities, including anti-microbial [11], anti-inflammatory [12], anti-obesity [13,14], anti-cancer [15], and anti-oxidant activities [12,16,17]. Previous phytochemical investigations of *M. alba* fruit have reported a variety of bioactive secondary metabolites such as chlorogenic acid, ferulic acid, protocatechuic acid, apigenin, quercetin, and rutin [18]. In our ongoing endeavor to find bioactive products from diverse natural resources [19,20,21,22], we have carried out chemical investigations of many natural materials to identify bioactive compounds exhibiting protective effects against cisplatin-induced nephrotoxicity. As a result, we have identified several kidney-protective phytochemicals, such as ginsenoside Rb1 from *Panax ginseng* [23], ergosterols from the fruiting bodies of the mushroom *Pleurotus cornucopiae* [24], and flavonoids from peat moss *Sphagnum palustre* [25]. Recently, we also identified butyl pyroglutamate, a renoprotective compound, from *M. alba* fruit [26]. Its renoprotection was mediated by inhibition of MAPK protein expression and cleaved caspase-3 protein expression [26].

To extend our previous studies, we further investigated an ethanol extract of *M. alba* fruit to identify potential renoprotective compounds in the present study. Phytochemical analysis of the *M. alba* fruit extract led to the isolation of six phytosterols (**1**–**6**). Their structures were determined by detailed analyses of their nuclear magnetic resonance (NMR) spectroscopic and physical data as well as mass spectrometry (MS) data from liquid chromatography (LC)/MS analyses. Herein, we report the isolation and structural characterization of these six compounds along with their protective effects against cisplatin-induced cell death and their underlying mechanism of action in LLC-PK1 cells.

## 2. Materials and Methods

### 2.1. General Experimental Procedures

Optical rotations were measured using a Jasco P-1020 polarimeter (Jasco, Easton, MD, USA). Infrared (IR) spectra were recorded using a Bruker IFS-66/S FT-IR spectrometer (Bruker, Karlsruhe, Germany). Electrospray ionization (ESI) mass spectra were recorded using a Waters Micromass Q-Tof Ultima ESI-TOF mass spectrometer (Waters, New York, NY, USA). Nuclear magnetic resonance (NMR) spectra were recorded using a Bruker AVANCE III 700 NMR spectrometer operating at 700 MHz (^1^H) and 175 MHz (^13^C) (Bruker, Karlsruhe, Germany) with chemical shifts reported in parts per million (δ). Preparative HPLC used a Waters 1525 Binary HPLC pump with a Waters 996 Photodiode Array Detector (Waters Corporation, Milford, CT, USA). Semi-preparative HPLC was performed using a Shimadzu Prominence HPLC System with SPD-20A/20AV Series Prominence HPLC UV-Vis Detectors (Shimadzu, Tokyo, Japan). Silica gel 60 (Merck, 70–230 mesh and 230–400 mesh) and RP-C18 silica gel (Merck, 40–63 μm) were used for column chromatography. Merck precoated silica gel F254 plates and RP-18 F254s plates (Merck, Darmstadt, Germany) were used for thin layer chromatography (TLC). Spots were detected on TLC under UV light or by heating after spraying with anisaldehyde-sulfuric acid.

### 2.2. Plant Material, Extraction, and Isolation

Fruit from *M. alba* was collected in China in January 2014. A voucher specimen (MA 1414) of the material was identified by one of the authors (K.H. Kim) and placed in the herbarium of the School of Pharmacy, Sungkyunkwan University, Suwon, Korea. Dried *M. alba* fruit was processed using 70% aqueous ethanol and then evaporated in vacuo to obtain a crude brownish ethanol extract (1.4 kg). The ethanol extract was solvent-partitioned with hexane, CH_2_Cl_2_, EtOAc, and butanol three times to obtain four main fractions yielding 27.8, 85.3, 32.9, and 138.8 g, respectively. The methylene chloride (CH_2_Cl_2_)-soluble fraction was subjected to open silica gel column (230–400 mesh) chromatography and fractionated using a gradient solvent system of CH_2_Cl_2_–MeOH (50:1–1:1) to produce five fractions (A–E). Fraction B (2.3 g) was further fractionated by open RP-C18 silica gel column (230–400 mesh) chromatography using a gradient solvent system of methanol–water (MeOH-H_2_O) (7:3–1:0) to produce 11 subfractions (B1–B11). Four subfractions (B91–B94) were acquired from subfraction B9 (398 mg) using a silica gel column (230–400 mesh) with a gradient solvent system of dichloromethane–methanol (CH_2_Cl_2_–MeOH) (50:1–1:1). Subfraction B91 (25 mg) was injected onto semi-preparative reversed-phase HPLC using 91% aqueous MeOH to obtain compounds **1** (6.0 mg, *t*_R_ = 42.0 min) and **2** (7.2 mg, *t*_R_ = 47.0 min). Subfraction B93 (38 mg) was separated utilizing semi-preparative reversed-phase HPLC eluted with 92% aqueous MeOH to obtain compounds **3** (4.0 mg, *t*_R_ = 51.5 min) and **4** (6.7 mg, *t*_R_ = 53.0 min). Fraction C (1.8 g) was fractionated using a silica gel column (230–400 mesh) and eluted with a gradient solvent system of CH_2_Cl_2_–MeOH (100:1–1:1) to obtain seven subfractions (C1–C7). Three subfractions (C71–C73) were acquired from subfraction C7 (330 mg) using a silica gel column (230–400 mesh) with a gradient solvent system of CH_2_Cl_2_–MeOH (30:1–1:1). Compounds **5** (6.3 mg, *t*_R_ = 32.5 min) and **6** (3.1 mg, *t*_R_ = 52.5 min) were purified from subfraction C72 (120 mg) using semi-preparative reversed-phase HPLC eluted with 85% aqueous MeOH.

### 2.3. Cell Culture and Cell Viability Assay

LLC-PK1 cells and kidney epithelial cells from pigs were purchased from the American Type Culture Collection (ATCC, Manassas, VA, USA). These cells were grown at 37 °C in a humidified atmosphere incubator with 5% CO_2_ in air using Dulbecco’s modified eagle medium (ATCC) supplemented with 1% penicillin/streptomycin, 10% fetal bovine serum (Invitrogen, Grand Island, NY, USA), and 4 mM l-glutamine. These cells were seeded into 96-well culture plates at a density of 1 × 10^4^ cells/mL. After 24 h, cells were pretreated with 2.5, 5, 10, 25, and 50 μM of test samples for 2 h at 37 °C. Next, 25 μM cisplatin was added to cells. After incubation for 24 h at 37 °C, cell viability was measured using an EZ-Cytox assay kit (Daeillab Service, Seoul, South Korea) according to the method described in a previous study [26].

### 2.4. Image-Based Cytometric Assay

Annexin V Alexa Fluor 488 staining was performed to determine the percentage of apoptotic cells. Briefly, cells were seeded in six-well plates at a density of 4 × 10^5^ cells/mL. After 24 h, cells were pretreated with 2.5 and 5 μM compound **1** for 2 h at 37 °C. Next, 25 μM cisplatin was added to cells. After incubation for 24 h at 37 °C, cells were stained with Annexin V Alexa Fluor 488 (Invitrogen, Temecula, CA, USA). The percentage of apoptotic cells was analyzed using a Tali image-based cytometer (Invitrogen, Temecula, CA, USA) according to the method described in a previous study [26].

### 2.5. Western Blotting Analysis

Cells were seeded into six-well plates at a density of 4 × 10^5^ cells/mL. After 24 h, cells were pretreated with 2.5 and 5 μM compound **1** for 2 h at 37 °C. Next, 25 μM cisplatin was added to cells. After incubation for 24 h at 37 °C, Western blot analysis was performed according to a previously described method [26]. The same amount of protein was transferred to Immobilon-P (PVDF) transfer membranes (Millipore, Bedford, MA, USA) from a precast 4–15% Mini-PROTEAN TGX gel (Bio-Rad, Hercules, CA, USA). The membranes were then incubated with primary antibodies and secondary antibodies. Primary and secondary antibodies were purchased from Cell Signaling Technology, Inc. (Beverly, MA, USA). The primary antibodies used in this study were phospho-p38 (1:1000 dilution), p38 (1:1000 dilution), phospho-JNK (1:1000 dilution), JNK (1:1000 dilution), cleaved caspase-3 (1:1000 dilution), and GAPDH (1:1000 dilution).

### 2.6. Statistical Analysis

All data, including cell viability, percentage of apoptotic cells, and protein expression, are presented as average value and standard deviation (SD). All assays were performed in triplicate and repeated at least thrice. In this study, only a small number of repetitions for each cell experiment were included. Thus, a non-parametric analysis method was adopted for the statistical analysis. The Kruskal–Wallis test was used for the statistical analysis of each variable. The SPSS statistical package (IBM SPSS Statistics version 21, Boston, MA, USA) was used for all analyses. Statistical significance was considered at *p* < 0.05.

## 3. Results

### 3.1. Isolation and Identification of Compounds

**Figure 1:**
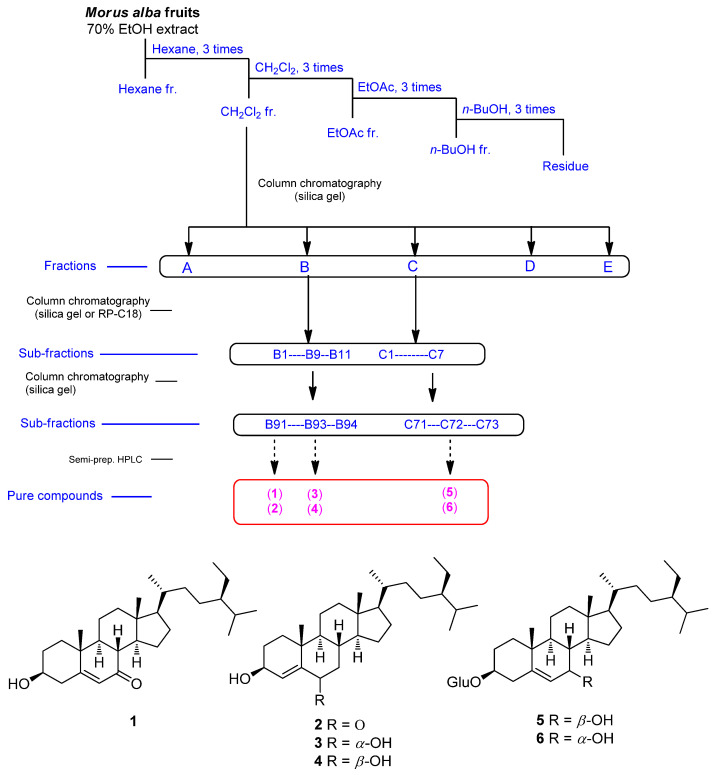
Separation scheme and chemical structures of compounds **1**–**6**.

Dried and pulverized *M. alba* fruit was extracted with 70% ethanol three times at room temperature. Aqueous ethanol was evaporated in vacuo to obtain the ethanol extract. To discover bioactive compounds, we performed solvent partitioning on the ethanol extract using hexane, dichloromethane (CH_2_Cl_2_), ethyl acetate (EtOAc), and *n*-butanol (*n*-BuOH). Repetitive fractionation and purification of open column chromatography and semi-preparative high-performance liquid chromatography (HPLC) on the CH_2_Cl_2_-soluble fraction led to the isolation of six phytosterols (**1**–**6**) (Figure 1). The structures of these isolated compounds (Figure 1) were elucidated as 7-ketositosterol (**1**) [27], stigmast-4-en-3β-ol-6-one (**2**) [28], (3β,6α)-stigmast-4-ene-3,6-diol (**3**) [29], stig-mast-4-ene-3β,6β-diol (**4**) [30], 7β-hydroxysitosterol 3-O-β-d-glucoside (**5**) [31], and 7α-hydroxysitosterol 3-O-β-d-glucoside (**6**) [31] by analyzing their physical and NMR spectroscopic data (Figures S1–S12) compared with those reported in previous studies and data from LC/MS analysis.

### 3.2. Compounds Isolated from M. alba Fruit Inhibit Cisplatin-Induced Death of LLC-PK1 Cells

**Figure 2:**
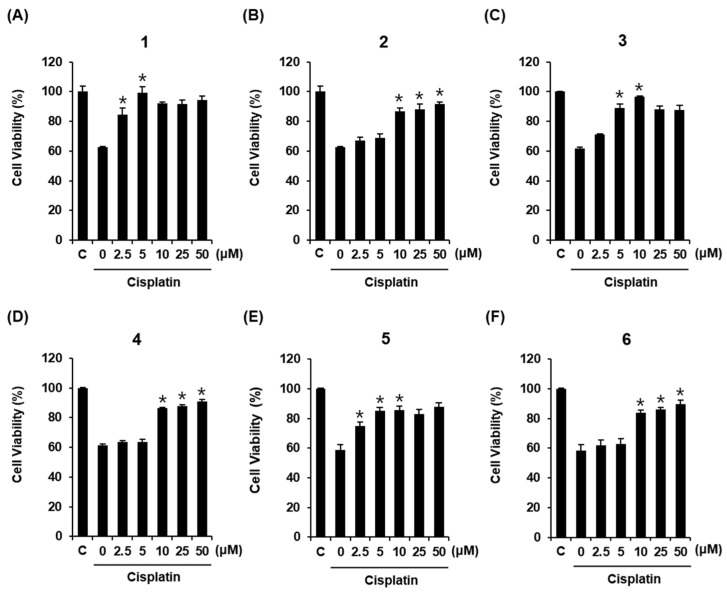
Protective effects of (**A**) 7-ketositosterol (**1**), (**B**) stigmast-4-en-3β-ol-6-one (**2**), (**C**) (3β,6α)-stigmast-4-ene-3,6-diol (**3**), (**D**) stig-mast-4-ene-3β,6β-diol (**4**), (**E**) 7β-hydroxysitosterol 3-O-β-D-glucoside (**5**), and (**F**) 7α-hydroxysitosterol 3-O-β-D-glucoside (**6**) on LLC-PK1 cells exposed to 25 μM of cisplatin for 24 h by MTT assay. Control cells were treated with vehicle only (mean ± SD of n = 3 replicates, * *p* < 0.05 compared with the control).

Cisplatin-induced LLC-PK1 cell death was used to examine the renoprotective effects of compounds isolated from *M. alba* fruit. Treatment of LLC-PK1 cells with 25 μM cisplatin for 24 h caused a 62.58% ± 0.47% reduction in cell viability compared with untreated controls (Figure 2A). All compounds displayed protective effects against cisplatin-induced damage in LLC-PK1 cells. The LLC-PK1 cell viability reduced by 25 μM cisplatin increased to 84.4% ± 4.33% and 99.09% ± 4.25% after co-treatment with compound **1** at 2.5 μM and 5 μM, respectively (Figure 2A). The LLC-PK1 cell viability reduced by 25 μM cisplatin increased to 86.68% ± 2.37%, 88.28% ± 3.24%, and 91.82% ± 1.11% after co-treatment with compound **2** at 10, 25, and 50 μM, respectively (Figure 2B). The LLC-PK1 cell viability reduced by 25 μM cisplatin increased to 89.15% ± 2.71% and 96.71% ± 0.31% after co-treatment with compound **3** at 5 and 10 μM, respectively (Figure 2C). The LLC-PK1 cell viability reduced by 25 μM cisplatin increased to 86.31% ± 0.73%, 87.59% ± 1.12%, and 90.85% ± 1.22% after co-treatment with compound **4** at 10, 25, and 50 μM, respectively (Figure 2D). The LLC-PK1 cell viability reduced by 25 μM cisplatin increased to 74.71% ± 2.92%, 85.25% ± 2.31%, and 85.63% ± 2.69% after co-treatment with compound **5** at 2.5, 5, and 10 μM, respectively (Figure 2E). The LLC-PK1 cell viability reduced by 25 μM cisplatin increased to 86.12% ± 1.21%, 89.68% ± 2.67%, and 92.47% ± 4.02% after co-treatment with compound **6** at 10, 25, and 50 μM, respectively (Figure 2F). The best protective effect on LLC-PK1 cells exposed to 25 μM cisplatin was observed for treatment with 5 μM of compound **1**. Therefore, compound **1** was selected for subsequent analysis.

### 3.3. Compound ***1*** Inhibits Cisplatin-Induced Apoptosis in LLC-PK1 Cells

**Figure 3:**
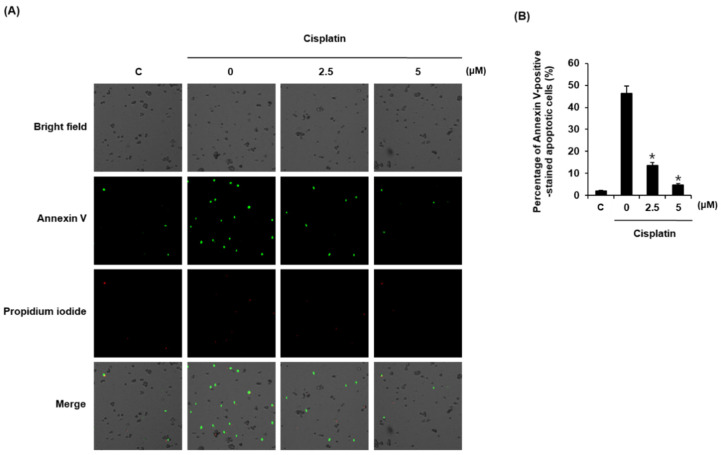
Protective effects of compound **1** on apoptosis of LLC-PK1 cells exposed to 25 μM cisplatin for 24 h assessed by image-based cytometric assay. (**A**) Representative images for apoptosis detection (green fluorescence); magnification: 4×; (**B**) Percentage of Annexin-V-positive stained apoptotic cells. Control cells were treated with vehicle only (mean ± SD of n = 3 replicates, * *p* < 0.05 compared with the control).

We evaluated the effects of compound **1** on cisplatin-induced apoptotic cell death using Annexin V Alexa Fluor 488 staining. As shown in Figure 3A, apoptotic cells were stained with Annexin V Alexa Fluor 488 (green fluorescence). The percentage of apoptotic cells was increased by 25 μM cisplatin from 2.13% ± 0.19% to 46.41% ± 3.21%, whereas it was decreased by 13.74% ± 1.31% and 4.86% ± 0.49% when cells were pretreated with 10 μM and 25 μM of compound **1**, respectively (Figure 3B).

### 3.4. Compound ***1*** Inhibits Expression Levels of p38, JNK, and Cleaved Caspase-3 in Cisplatin-Treated LLC-PK1 Cells

**Figure 4:**
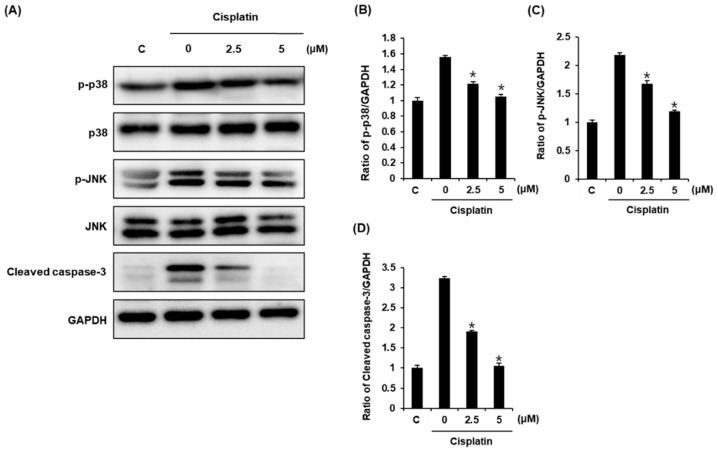
Protective effects of compound **1** on apoptosis of LLC-PK1 cells exposed to 25 μM cisplatin for 24 h as assessed by a Western blot analysis. (**A**) Expression levels of phospho-p38 (p-p38), p38, phospho-c-Jun N-terminal kinase(p-JNK), JNK, and cleaved caspase-3. (**B**–**D**) Each bar graph represents densitometric quantification of Western blot bands. Control cells were treated with vehicle only (mean ± SD of n = 3 replicates, * *p* < 0.05 compared with the control).

We also evaluated the possible molecular mechanisms of compound **1**, focusing on p38, JNK, and cleaved caspase-3 using a Western blot analysis. Treatment with 25 μM cisplatin increased the expression levels of phosphorylated p38, phosphorylated JNK, and cleaved caspase-3. However, the expression levels of all these proteins in LLC-PK1 cells were decreased by treatment with 2.5 and 5 μM compound **1** in a dose-dependent manner (Figure 4A). Bar graphs show the expression levels of phosphorylated p38, phosphorylated JNK, and cleaved caspase-3 normalized to glyceraldehyde 3-phosphate dehydrogenase (GAPDH) (Figure 4B–D).

## 4. Discussion

Many drugs, including antifungal agents, anti-retroviral drugs, aminoglycoside antibiotics, and anticancer drugs, are known to cause nephrotoxicity [32]. Various assays have been used to assess the protective effects of plant extracts and plant-derived natural products against drug-induced cytotoxicity in kidney cells. The primary assay to identify an effective substance is based on measurement of cell viability. In the present study, we identified cell-protective compounds from *M. alba* fruit using the EZ-Cytox assay to measure the metabolic activities of cells in the presence of cisplatin. All compounds displayed protective effects against cisplatin-induced LLC-PK1 cell damage, improving cisplatin-induced cytotoxicity to more than 80% of the control value. Compound **1** displayed the best effect at a relatively low concentration. The LLC-PK1 cell viability that was reduced by 25 μM cisplatin to 60% increased to nearly 100% after co-treatment with 5 μM compound **1**. In our previous study, 10 μM butyl pyroglutamate isolated from *M. alba* fruit improved the cell viability by 83%, which was more effective than N-acetylcysteine [33]. N-acetylcysteine has been used as a positive control in cisplatin-induced renal toxicity studies [34,35].

**Figure 5:**
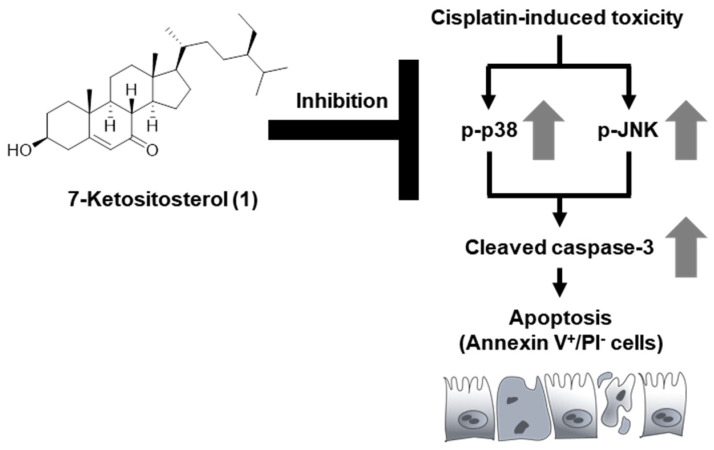
Schematic pathway for the potential role of 7-ketositosterol (**1**) in renoprotective effects.

Oxidative stress, apoptosis, and inflammation are three major mechanisms underlying cisplatin-induced cytotoxicity. Among these, the most well-known mechanism is the apoptosis pathway [35]. It is known that cisplatin-induced apoptotic cell death in renal tubular cells is associated with both mitochondrial-mediated and death-receptor-mediated pathways [36]. Both these pathways ultimately induce apoptosis through caspase-3 activation [37]. Additionally, it has been shown that JNK and p38 regulate tumor necrosis factor-α (TNF-α), which plays an important role in cisplatin-induced apoptosis [38,39]. In the present study, compound **1** had a protective effect against apoptotic cell death. This result is consistent with the improved cell viability of compound-**1**-treated cells. The protective effect of compound **1** on LLC-PK1 cells might be partly due to inhibition of apoptosis by cisplatin. In addition, treatment with cisplatin increased the expression levels of phosphorylated p38, phosphorylated JNK, and cleaved caspase-3, whereas these expression levels were decreased in a dose-dependent manner by treatment of LLC-PK1 cells with compound **1**. These observations indicated that compound **1** inhibited apoptosis through the inhibition of phosphorylated JNK and p38 as well as the inhibition of the expression level of cleaved caspase-3 (Figure 5). Therefore, the anti-apoptotic effect might be responsible for the protective effect of compound **1** against cisplatin-induced cell death.

## 5. Conclusions

In summary, as part of an ongoing research project to discover bioactive natural products [40,41,42,43,44,45], we identified renoprotective phytosterols from the fruit of the mulberry tree (*M. alba*) that ameliorated cisplatin-induced cytotoxicity. All compounds displayed protective effects against cisplatin-induced damage in LLC-PK1 cells. Compound **1** displayed the best effect at a relatively low concentration. In addition, we demonstrated that compound **1** blocked cisplatin-induced LLC-PK1 cell apoptosis by inhibiting expression levels of phosphorylated p38, phosphorylated JNK, and cleaved caspase-3. However, additional detailed mechanisms responsible for the renoprotective effects of compound **1** need to be studied to support the potential of 7-ketositosterol (**1**) as an adjuvant candidate for treating cisplatin-induced nephrotoxicity.

## Data Availability

The data presented in this study are available in article and supplementary material.

## Supplementary Materials

The following are available online at https://www.mdpi.com/article/10.3390/plants10112481/s1, Figure S1: ^1^H NMR spectrum of compound **1** (in CDCl_3_), Figure S2: ^13^C NMR spectrum of compound **1** (in CDCl_3_), Figure S3: 1H NMR spectrum of compound **2** (in CDCl3), Figure S4: ^13^C NMR spectrum of compound **2** (in CDCl_3_), Figure S5: ^1^H NMR spectrum of compound **3** (in CD_3_OD), Figure S6: ^13^C NMR spectrum of compound **3** (in CD_3_OD), Figure S7: ^1^H NMR spectrum of compound **4** (in CDCl_3_), Figure S8: ^13^C NMR spectrum of compound **4** (in CDCl_3_), Figure S9: ^1^H NMR spectrum of compound **5** (in CD_3_OD), Figure S10: ^13^C NMR spectrum of compound **5** (in CD_3_OD), Figure S11: ^1^H NMR spectrum of compound **6** (in CD_3_OD), Figure S12: ^13^C NMR spectrum of compound **6** (in CD_3_OD).
