# Supplementary material for: Identification of Renoprotective Phytosterols from Mulberry (Morus alba) Fruit against Cisplatin-Induced Cytotoxicity in LLC-PK1 Kidney Cells
Source: Plants (Basel). 2021 Nov 17;10(11):2481. doi: 10.3390/plants10112481 (PMC8623081; doi:10.3390/plants10112481)
Supplement: Supplementary file 1 [file plants-10-02481-s001.zip › plants-1453501-supplementary.pdf]

Supplementary Materials

Identification of Renoprotective Phytosterols from Mulberry (*Morus alba*) Fruit against Cisplatin-Induced Cytotoxicity in LLC-PK1 Kidney Cells

Dahae Lee [1,†], Seoung Rak Lee [2,†], Bang Ju Park [3], Ji Hoon Song [4], Jung Kyu Kim [5], Yuri Ko [6], Ki Sung Kang [1,*] and Ki Hyun Kim [2,6,*]

[1] College of Korean Medicine, Gachon University, Seongnam 13120, Korea; pjsldh@gachon.ac.kr
[2] School of Pharmacy, Sungkyunkwan University, Suwon 16419, Korea; seungrak@princeton.edu
[3] Department of Electronic Engineering, Gachon University, Seongnam 13120, Korea; sooyong1320@gachon.ac.kr
[4] Jeju Institute of Korean Medicine, Jeju 63309, Korea; jhsong@jikom.or.kr
[5] School of Chemical Engineering, Sungkyunkwan University, Suwon 16419, Korea; legkim@skku.edu
[6] Department of Biological Chemistry and Molecular Pharmacology, Harvard Medical School, Boston, MA 02115, USA; koyr0120@gmail.com
* Correspondence: kkang@gachon.ac.kr (K.S.K.); khkim83@skku.edu (K.H.K.); Tel.: +82-31-750-5402 (K.S.K.); +82-31-290-7700 (K.H.K.)
† These authors contributed equally to this study.

Figure S1. ^{1}H NMR spectrum of compound **1** (in $CDCl_3$).

Figure S2. ^{13}C NMR spectrum of compound **1** (in $CDCl_3$).

Figure S3. ^{1}H NMR spectrum of compound **2** (in $CDCl_3$).

Figure S4. ^{13}C NMR spectrum of compound **2** (in $CDCl_3$).

Figure S5. ^{1}H NMR spectrum of compound **3** (in CD_3OD).

Figure S6. ^{13}C NMR spectrum of compound **3** (in CD_3OD).

Figure S7. ^{1}H NMR spectrum of compound **4** (in $CDCl_3$).

Figure S8. ^{13}C NMR spectrum of compound **4** (in $CDCl_3$).

Figure S9. ^{1}H NMR spectrum of compound **5** (in CD_3OD).

Figure S10. ^{13}C NMR spectrum of compound **5** (in CD_3OD).

Figure S11. ^{1}H NMR spectrum of compound **6** (in CD_3OD).

Figure S12. ^{13}C NMR spectrum of compound **6** (in CD_3OD).

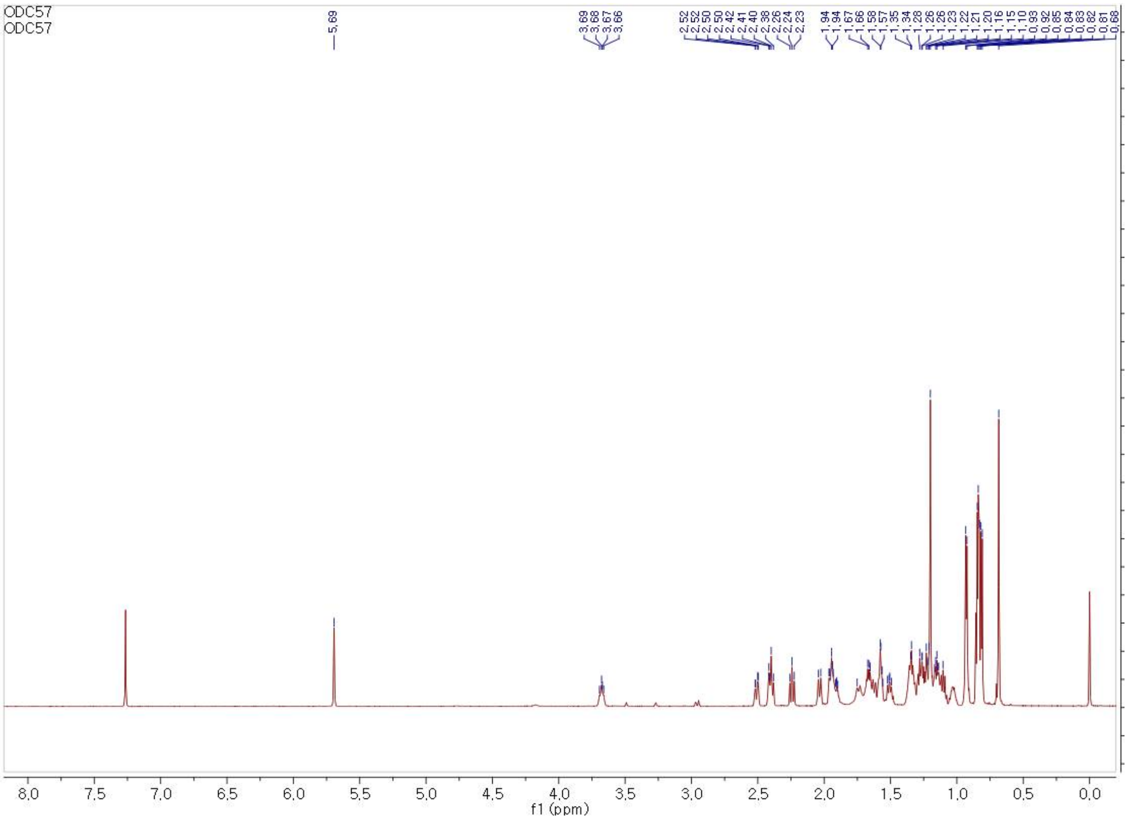

Figure S1. ^{1}H-NMR spectrum of compound **1** (in $CDCl_3$).

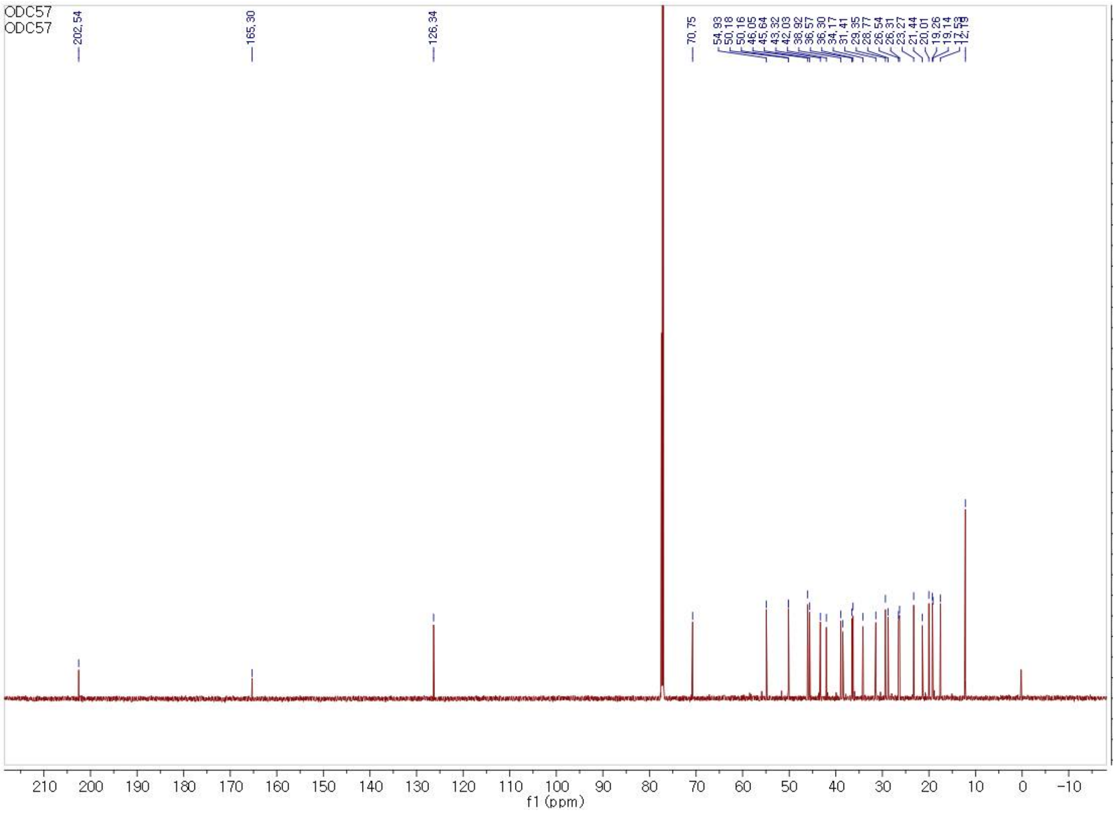

Figure S2. ^{13}C-NMR spectrum of compound **1** (in $CDCl_3$).

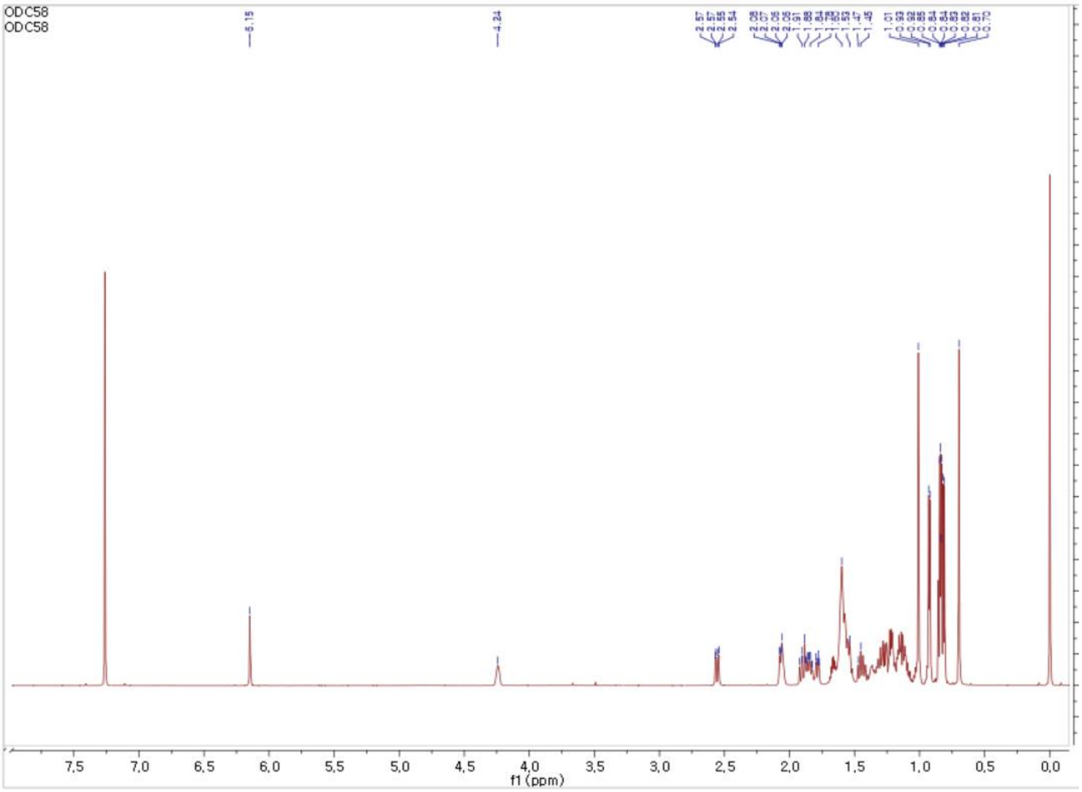

Figure S3. ^{1}H-NMR spectrum of compound **2** (in $CDCl_3$).

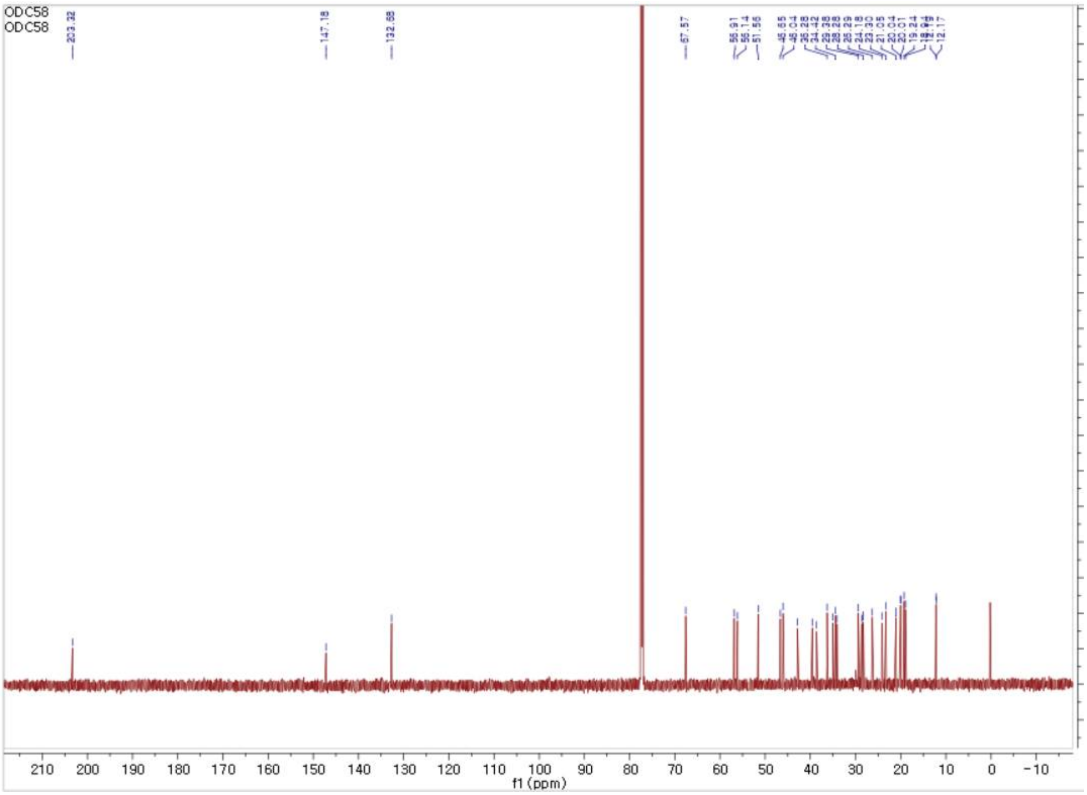

Figure S4. ^{13}C-NMR spectrum of compound **2** (in $CDCl_3$).

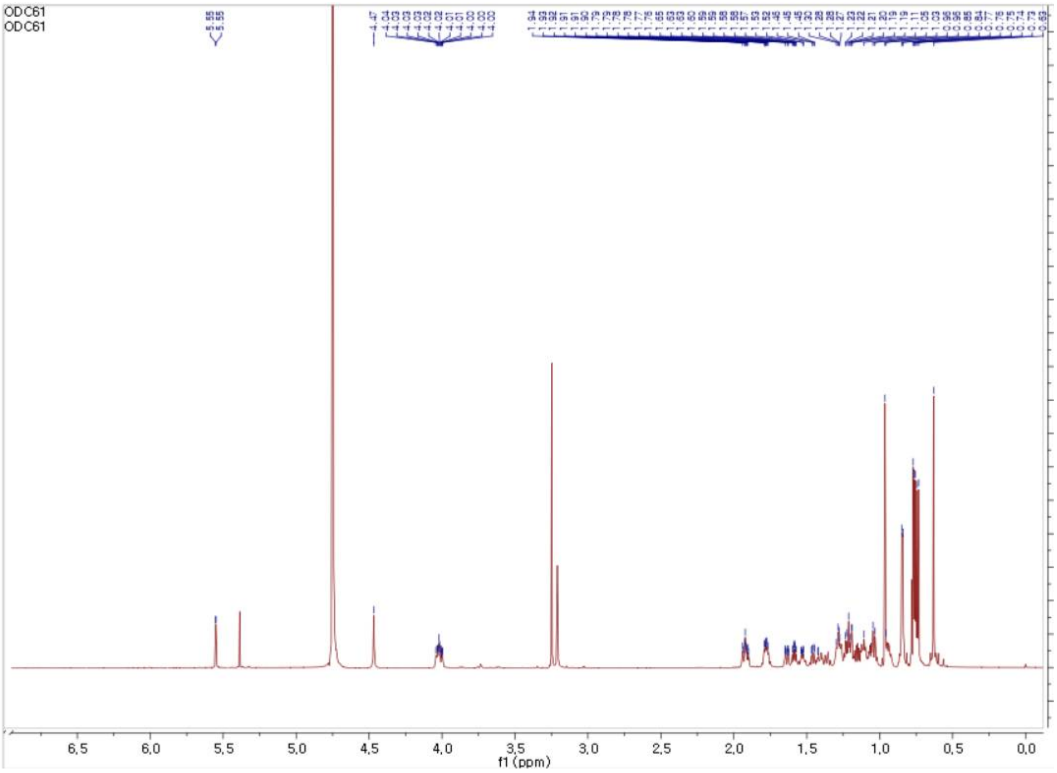

Figure S5. ^{1}H-NMR spectrum of compound **3** (in CD_3OD).

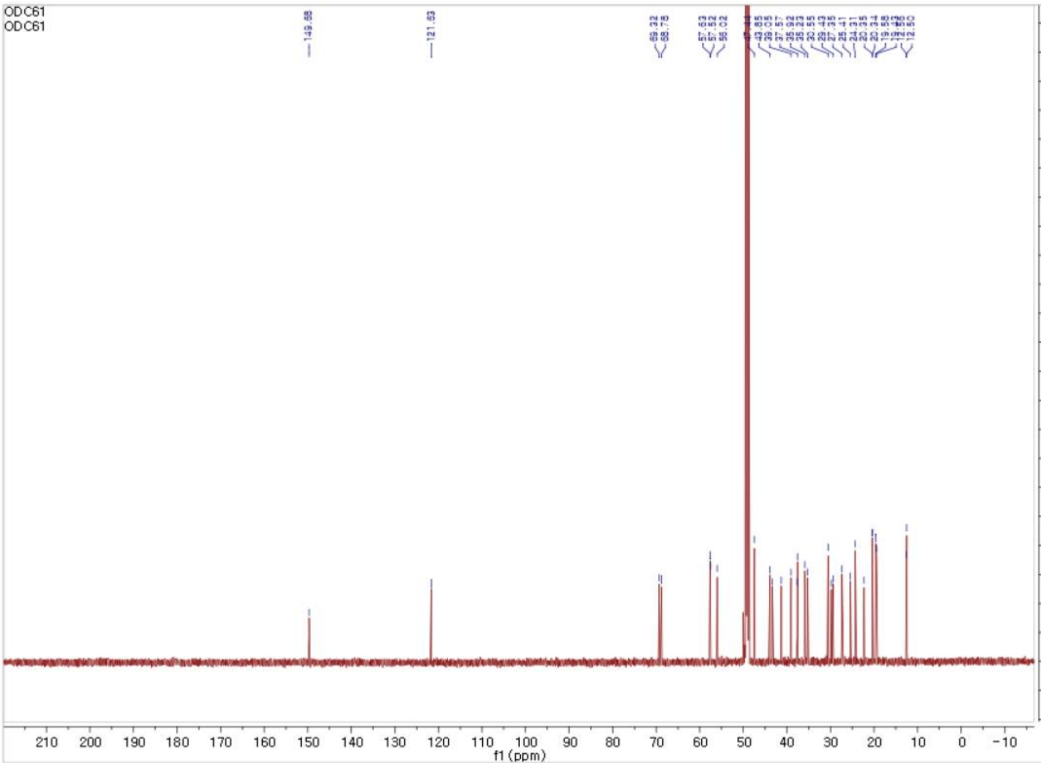

Figure S6. ^{13}C-NMR spectrum of compound **3** (in CD_3OD).

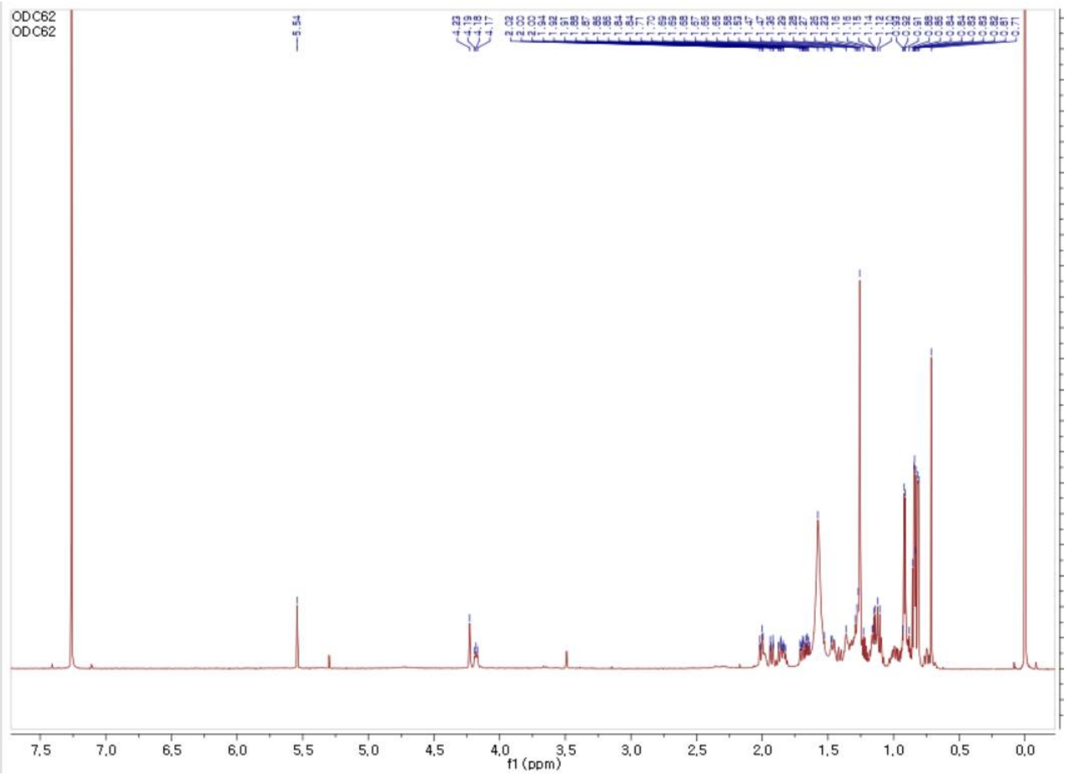

Figure S7. ^{1}H-NMR spectrum of compound **4** (in $CDCl_3$).

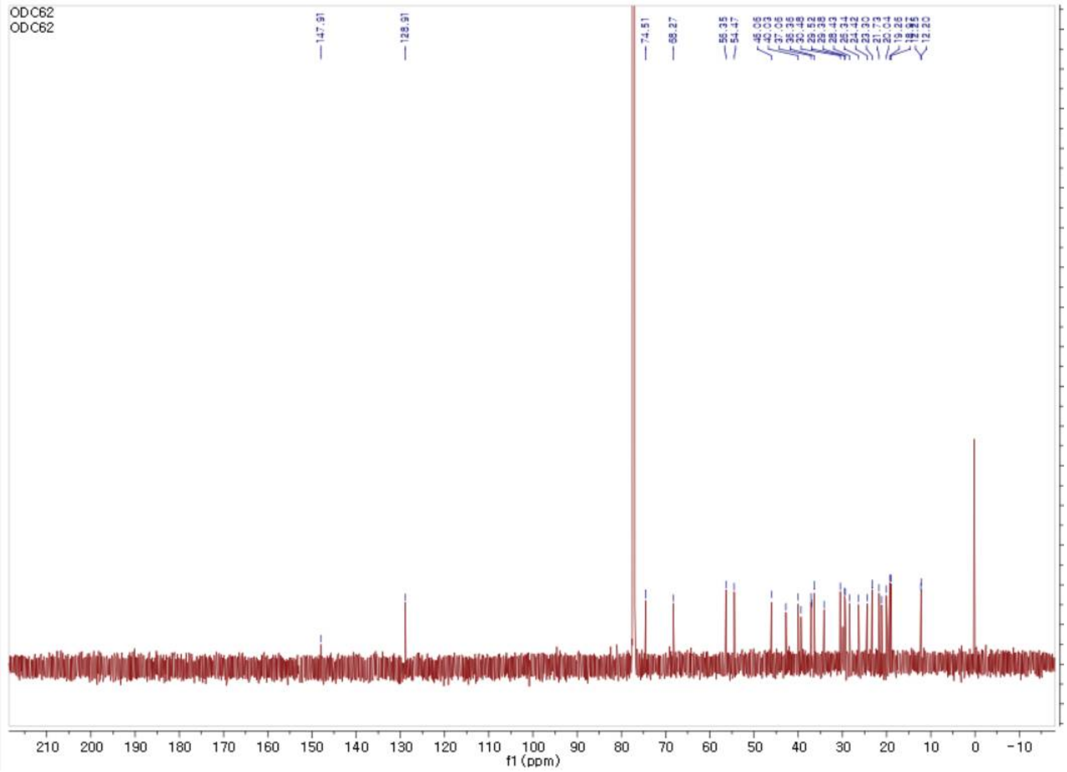

Figure S8. ^{13}C-NMR spectrum of compound **4** (in $CDCl_3$).

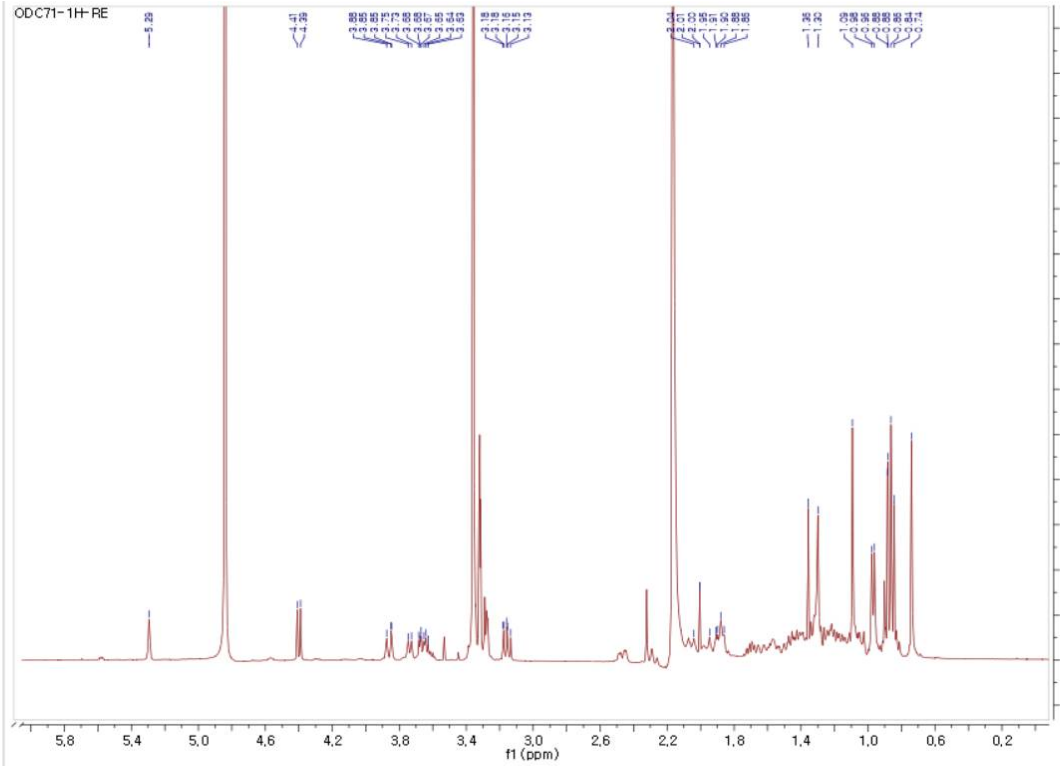

Figure S9. ^{1}H-NMR spectrum of compound **5** (in CD_3OD).

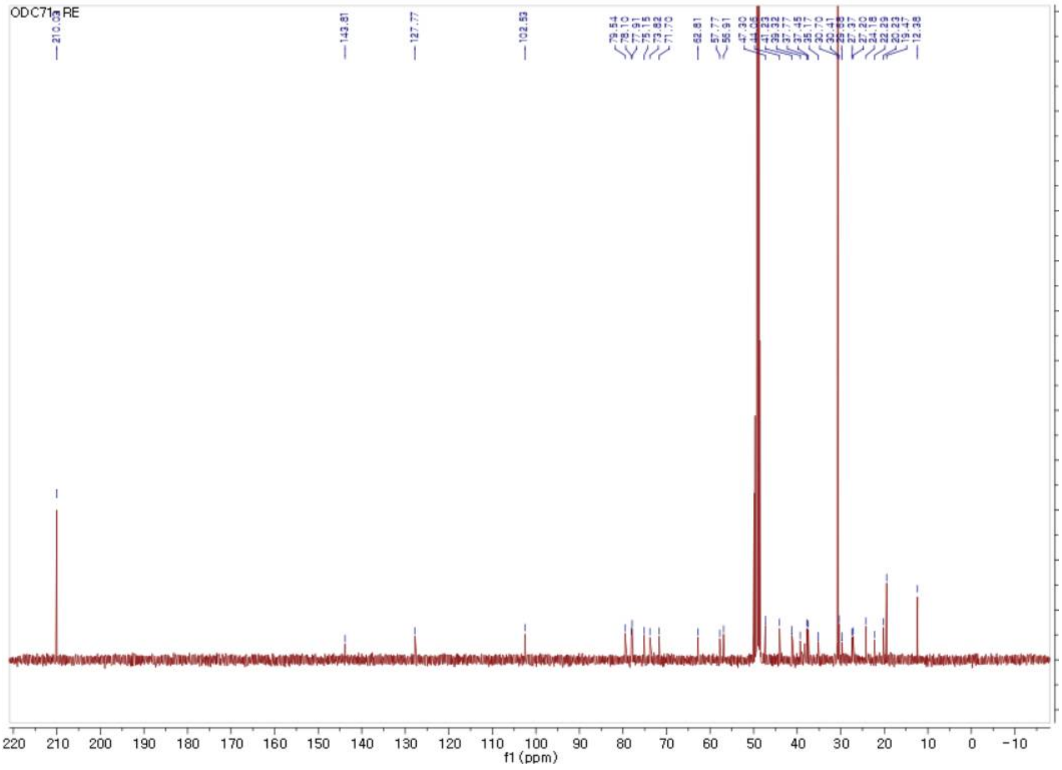

Figure S10. ^{13}C-NMR spectrum of compound **5** (in CD_3OD).

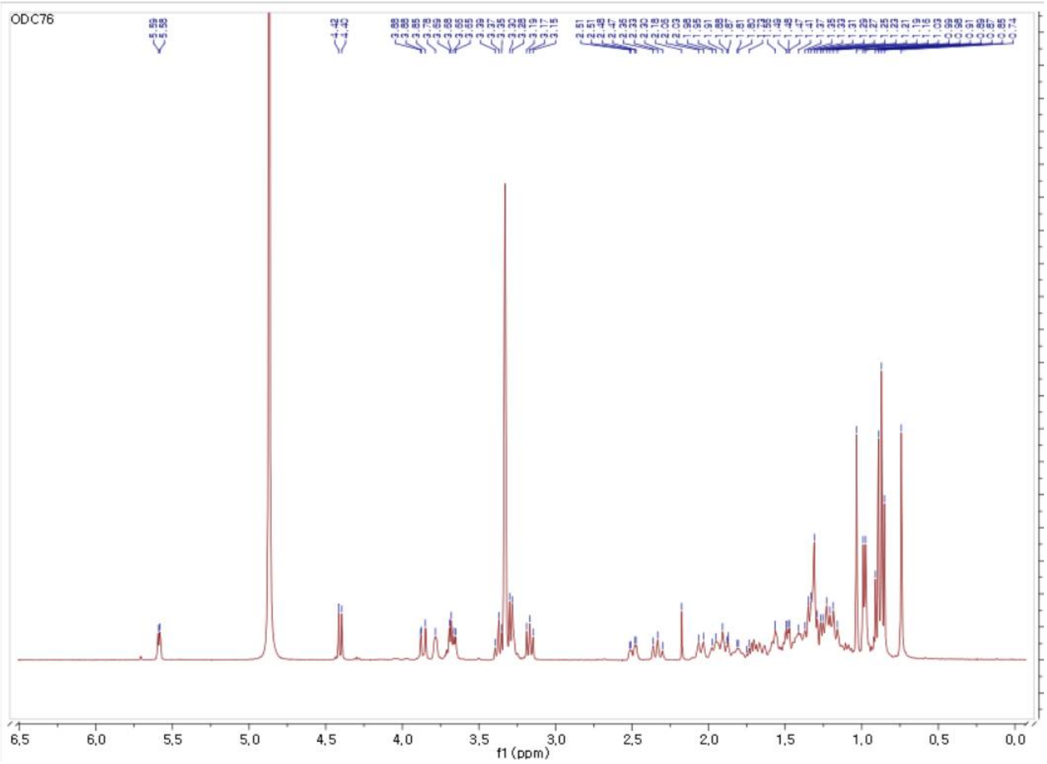

Figure S11. ^{1}H-NMR spectrum of compound **6** (in CD_3OD).

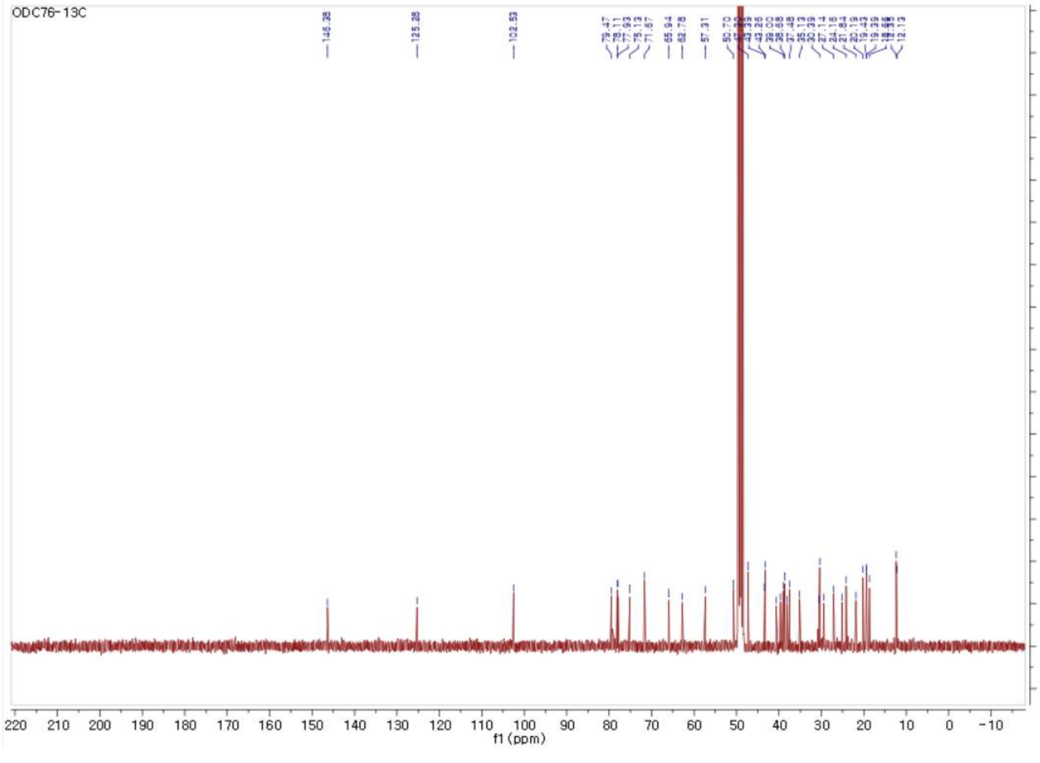

Figure S12. ^{13}C-NMR spectrum of compound **6** (in CD_3OD).
